# Ecosystem-Based Incorporation of Nectar-Producing Plants for Stink Bug Parasitoids

**DOI:** 10.3390/insects8030065

**Published:** 2017-06-24

**Authors:** Glynn Tillman

**Affiliations:** Crop Protection & Management Research Laboratory, USDA, ARS, Tifton, GA 31793, USA; Glynn.Tillman@ars.usda.gov; Tel.: +1-229-387-2375

**Keywords:** parasitoids, nectar-provision, biocontrol, stink bugs, pollination, conservation

## Abstract

Adult parasitoids of pest insects rely on floral resources for survival and reproduction, but can be food-deprived in intensively managed agricultural systems lacking these resources. Stink bugs are serious pests for crops in southwest Georgia. Provisioning nectar-producing plants for parasitoids of stink bugs potentially can enhance biocontrol of these pests. Knowledge of spatial and temporal availability and distribution of stink bugs in host plants is necessary for appropriate timing and placement of flowering plants in agroecosystems. Stink bugs move between closely associated host plants throughout the growing season in response to deteriorating suitability of their host plants. In peanut-cotton farmscapes, stink bugs develop in peanut, and subsequently the adults disperse into adjacent cotton. Parasitism of *Nezara viridula* (L.) adults by *Trichopoda pennipes* (F.) at the peanut-cotton interface was significantly higher in cotton with a strip of milkweed or buckwheat between the two crops than in cotton alone. Milkweed and buckwheat also provided nectar to a wide range of insect pollinators. Monarch butterflies fed on milkweed. When placed between peanut and cotton, a strip of soybean was an effective trap crop for cotton, reducing economic damage. Incorporation of buckwheat near soybean enhanced parasitism of *Euschistus servus* (Say) eggs by *Telenomus podisi* Ashmead in cotton. In conclusion, nectar provision enhances biocontrol of stink bugs, acts together with other management tactics for stink bug control, and aids in conservation of natural enemies, insect pollinators, and the monarch butterfly.

## 1. Introduction

Many adult parasitoids of pest insects rely on floral food resources for survival and reproduction. Consumption of floral resources by parasitoids has been directly and indirectly confirmed. Visual observation of nectar-feeding in the field has been reported for many parasitoid species [1,2,3]. Gut sugar analyses have been applied to examine sugar-feeding by parasitoids in the field. High-performance liquid chromatography (HPLC) analyses demonstrated that over 85% of field-caught *Diadegma insulare* (Cresson) (Hymenoptera: Ichneumonidae), a parasitoid of *Plutella xylostella* (L.), had fed upon sugars [4]. In another study, analysis of sugar content in parasitoids demonstrated that adults collected adjacent to a flowering field border had higher levels of sugar compared with freshly emerged individuals, indicating that the parasitoids consumed sugars in the field [5]. Modern agricultural systems that depend on mechanical cultivation and chemical pesticides for insect control have resulted in a lack of floral resources in agroecosystems. Parasitoids in these agroecosystems can be severely food-deprived. For instance, *Meteorus autographae* Muesebeck (Hymenoptera: Braconidae) collected from cotton fields bordered by vegetation lacking in suitable sugar sources were sugar-limited [6]. Clearly, provision of floral resources for adult parasitoids of pest insects is an important aspect of habitat management for conservation biological control in agroecosystems.

Nectar-provision must be guided by the context of ecological principles for management of insect pests in agroecosystems. The position and size of a source of insects in an agroecosystem can determine colonization patterns of insects in crops [7]. Seasonal succession patterns of annual, perennial, shrub, and/or tree sources of insects can influence insect species composition and timing of colonization [8]. Thus, we must understand the biology and ecology of insects in an agroecosystem to design an effective spatial and temporal arrangement of a nectar-provision habitat to improve biological control.

### 1.1. Value of Feeding on Floral Nectar

Feeding on floral nectar has several positive effects on adult parasitoids, including increasing adult longevity, fecundity, and searching activity. Under laboratory conditions, parasitoids with access to flower nectar live longer and are more fecund than those with only access to water. For instance, alyssum (*Lobularia maritima* L.) and buckwheat (*Fagopyrum esculentum* Moench) nectar increased longevity and fecundity of the parasitoid *Gonatocerus ashmeadi* Girault (Hymenoptera: Mymaridae) compared to water [9]. Similarly, sugar sources increased longevity and fecundity of *Aphidius rhopalosiphi* De Stefani-Perez and *Diaeretiella rapae* McIntosh (Hymenoptera: Aphidiidae) [10].

Several field studies have shown that availability of flowers increases longevity and fecundity of parasitoids compared to areas which lack such resources. Females of *D. insulare* in cabbage plots with bordering flowering buckwheat had higher longevity, and likely fecundity, than females from plots without buckwheat, and ingestion of nectar correlated with longevity [11]. In an earlier field cage study, nectar feeding by *D. insulare* on wild mustard (*Brassica kaber* (D.C.) Wheeler), yellow rocket (*Barbarea vulgaris* R. Br.), and wild carrot (*Daucus carota* L.) resulted in longevity and fecundity equal to feeding on honey water [12]. In a study with large cages of Brussel sprouts with and without a pot of nectar-producing buckwheat, *Diadegma semiclausum* Helen (Hymenoptera: Ichneumonidae) females with access to nectar parasitized more *P. xylostella* larvae compared to those without access to nectar [13]. Nectar also increased the average reproductive lifespan of the parasitoids from 1.2 to 28 days. Longevity of *Copidosoma koehleri* Blanchard (Hymenoptera: Encyrtidae) was greater for adults caged on flowering plants of dill (*Anethum graveolens* L.), borage (*Borago officinalis* L.), or coriander (*Coriandrum sativum* L.) than those provided with only water [14]. 

Some studies have demonstrated an increase in parasitism of a pest in the presence of floral nectar. For instance, coriander and faba bean (*Vicia faba* L.) planted next to potato plots increased parasitism of the potato moth [*Phthorimaea operculella* (Zeller)] by *C. koehleri* [14]. When buckwheat was incorporated in the farmscape, parasitism by *Cotesia rubecula* (Marshall) (Hymenoptera: Braconidae) on imported cabbage worm [*Pieris rapae* (L.)] larvae was increased [15]. In a New York vineyard, English-Loeb et al. [16] showed that parasitism of *Erythroneura* leafhoppers was increased by 20% when flowering buckwheat was present between rows.

Feeding on floral resources can also influence searching behavior of parasitoids. For instance, the nectar of buckwheat increased searching time of the aphid parasitoid *D. rapae* by a factor of 40 compared with individuals provided with only water [17]. 

### 1.2. Biology and Ecology of Stink Bug Species and Their Parasitoids

Stink bugs (Hemiptera: Pentatomidae), including *Nezara viridula* (L.), *Chinavia hilaris* (Say), *Thyanta pallidovirens* (Stål), and *Euschistus* spp., i.e., *E. servus* (Say), *E. tristigmus* (Say), *E. conspersus* (Uhler), *E. ictericus* (L.), and *E. heros* (F.), are an overarching issue in all types of agriculture throughout the tropical and subtropical regions of the world [18,19]. The invasive brown marmorated stink bug, *Halyomorpha halys* (Stål), is a serious economic pest for orchard, row, and vegetable crops in the USA [20]. In farmscapes, one crop, or two or more closely associated crops, are bordered by woodland and/or non-woodland habitats. Provisioning nectar-producing plants for parasitoids of these stink bug species has the potential to enhance biocontrol of these pests in these farmscapes. Knowledge of spatial and temporal availability and distribution of stink bugs and their parasitoids in non-crop and crop host plant habitats, though, is necessary for appropriate timing and placement of nectar-producing plants in agroecosystems. 

Stink bugs move between closely associated host plants throughout the growing season in response to deteriorating suitability of their current host plants. Crop-to-crop dispersal of stink bug species has been reported for many cropping systems [21,22,23,24,25]. Although these studies are based on seasonal abundance or spatial distribution of stink bugs in adjoining crops, three mark-recapture studies have demonstrated direct movement of stink bugs from one crop to another [26,27,28]. For native species, as well as *H. halys*, stink bugs exhibit a pronounced edge effect during dispersal in crops [24,25,28,29,30,31,32,33,34]. Previous studies have indicated that stink bug adults move from non-crop host plants into nearby crop field edges [35,36,37,38,39]. For example, Ehler [40] determined that the first generation of *E. conspersus* Uhler developed on roadside weeds such as wild radish, *Raphanus sativus* L., and black mustard, *Brassica nigra* (L.) Koch, and the second generation developed in an adjacent tomato crop suggesting that after developing on roadside weeds, this stink bug dispersed into tomato. For *H. halys*, stink bug density was significantly higher in field edges adjacent to woodland habitats, suggesting that adults dispersed from non-crop hosts in woodlands into crops [34].

A diversity of parasitoid species parasitizes stink bug species in worldwide agroecosystems. *Trichopoda pennipes* (F.) parasitizes *N. viridula*, *C. hilaris*, and *H. halys* adults [41] while *Euthera tentatrix* Loew and *Cylindromyia binotata* (Bigot) (Diptera: Tachinidae) parasitize *E. servus* adults in North America [42,43]. *Trichopoda giacomellii* (Blanchard) is a South American adult parasitoid of *N. viridula* [41]. Several studies have been conducted to examine parasitism of eggs of native stink bug species and *H. halys* in a variety of crops, e.g., soybean, vegetables, alfalfa, tomato, peanut, corn, and cotton [20,43,44,45,46,47,48,49]. In each of these crops *Trissolcus basalis* (Wollaston) (Hymenoptera: Scelionidae) was the predominant egg parasitoid of *N. viridula* eggs, and *Telenomus podisi* Ashmead (Hymenoptera: Scelionidae), was the most prevalent parasitoid species emerging from *Euschistus* spp. and *H. halys* eggs. Interestingly, parasitism of stink bug eggs throughout the season indicates that egg parasitoids also exhibit crop-to-crop dispersal [48]. Clearly, design and implementation of nectar-provision for enhancement of natural enemies should be done in the context of the biology and ecology of stink bugs and their parasitoids in agroecosystems.

### 1.3. Stink Bugs and Their Parasitoids in Farmscapes in the Southeast USA

In the southeast USA, *C. hilaris*, *E. servus*, *E. tristigmus*, and *N. viridula* are the main serious pests of corn (*Zea mays* L.), peanut (*Arachis hypogaea* L.), *Bt* cotton (*Gossypium hirsutum* L.), and soybean [*Glycine max* (L.) Merr] in farmscapes. Corn and peanut are suitable hosts for *N. viridula* and *E. servus*, but not for *C. hilaris* [50]. In corn, *N. viridula* and *E. servus* feed on ears (i.e., fruit) which can result in economic damage [51,52]. Because stink bugs cannot feed on the underground fruit of peanut, they are not considered to be economic pests of this crop. Even so, *N. viridula* and *E. servus* oviposit on peanut leaves, and subsequent nymphs feed and develop on aboveground foliage [53]. In cotton, *C. hilaris*, *E. servus*, *E. tristigmus*, and *N. viridula* feed on developing seeds and lint of cotton, causing shedding of young bolls, yellowing of lint, yield reduction, and transmission of a strain of the bacterial pathogen *Pantoea agglomerans* which damages seeds and lint [54,55]. Currently, *H. halys* is considered an agricultural and nuisance pest in Georgia, primarily above the Coastal Plain Region [56].

Because of its early sowing, corn serves as a source of *N. viridula* and *E. servus* to later-planted adjacent crops, including peanut and fruiting cotton [57]. Thus, an edge effect in dispersal of *N. viridula* and *E. servus* into cotton occurs primarily at corn-cotton interfaces [33]. In peanut-cotton farmscapes, *N. viridula* and *E. servus* develop on peanut and then late-instar nymphs and young adults disperse to feed on newly-available cotton bolls [27]. An edge effect in dispersal of these two stink bug species, as well as *C. hilaris*, into cotton can be detected up to 8.2 m from the peanut-cotton interface [33]. Because peanut is not a host plant of *C. hilaris*, this stink bug likely disperses from early-season non-crop hosts across low-growing peanut into cotton.

Woodland borders in these farmscapes are comprised of planted pine (*Pinus* spp.), mixed hardwood-pine, forested wetlands, and cultivated and uncultivated pecan [*Carya illinoinensis* (Wangenh.) K. Koch.]. Non-woodland habitats include hay and cow pastures, ponds, and patches of grass and weeds in addition to paved and unpaved roadways. Preliminary results of a study on temporal and spatial distribution of stink bug trap capture across an 18 km^2^ landscape indicate that *C. hilaris*, *E. servus* and, to some extent, *E. tristigmus* primarily exist in farmscapes in which crops are bordered by habitats that harbor non-crop host plants. Black cherry (*Prunus serotina* Ehrh.), elderberry (*Sambucus nigra* subsp. *canadensis* [L.] R. Bolli), mimosa (*Albizia julibrissin* Durazz.), and pecan are non-crop reproductive hosts of stink bug species in wooded borders. Black cherry is an early-season host of *C. hilaris*, and mimosa is a mid-to-late-season host of this stink bug [37]. Pecan is an early-to-late season host of *E. servus*, *E. tristigmus*, and *C. hilaris* [58]. Tillman and Cottrell [59] demonstrated via a mark-recapture study that elderberry serves as a source of stink bugs dispersing into cotton. In late July and early August, as elderberry fruit senesce and cotton bolls, i.e., fruit, become available, *C. hilaris*, *E. servus*, and *E. tristigmus* begin dispersing from elderberry into cotton, resulting in an edge effect in cotton adjacent to woodlands. 

A variety of parasitoid species parasitizes the complex of stink bug species in farmscapes in the southeast USA. *Trichopoda pennipes* parasitizes *N. viridula* adults in each crop, and *C. hilaris* adults in cotton, while *Euthera tentatrix* Loew and *Cylindromyia binotata* (Bigot) (Diptera: Tachinidae) parasitize *E. servus* adults in each crop [53,60]. In north Georgia, *T. pennipes* also parasitizes *H. halys* adults [56]. Over a 10-yr period, parasitism of naturally-occurring eggs of *E. servus* and *C. hilaris* was assessed in crop and non-crop hosts [49]. Nine species of parasitoids, including seven scelionids and two eupelmids, parasitize *E. servus* eggs. *Telenomus podisi* Ashmead is the most prevalent parasitoid of *E. servus* eggs in each of three host plant habitats: early-season non-crop hosts in woodlands (i.e., black cherry and elderberry), an early-season crop (i.e., corn), and late-season crops (i.e., peanut, cotton, and soybean). *Trissolcus brochymenae* (Ashmead), *Trissolcus euschisti* (Ashmead), and *Trissolcus thyantae* Ashmead (Hymenoptera: Scelionidae) also parasitize *E. servus* eggs in each habitat. *Trissolcus edessae* Fouts is the most prevalent egg parasitoid of *C. hilaris* in woodlands and the only parasitoid of *C. hilaris* in late-season crops. In woodlands, *Anastatus reduvii* (Howard) (Hymenoptera: Eupelmidae) and *A. mirabilis* (Walsh & Riley) (Hymenoptera: Eupelmidae) parasitize *E. servus* and *C. hilaris* eggs primarily in woodlands, so parasitoid species diversity is relatively higher in this habitat. *Trissolcus basalis* (Wollaston) is the primary egg parasitoid of *N. viridula* eggs in crops [48]. In north Georgia, these native stink bug egg parasitoids also parasitize *H. halys* eggs [61].

The goal of this review is to present examples of on-farm application of nectar-provision for stink bug parasitoids based on the biology and ecology of stink bugs and their parasitoids in agroecosystems. The farmscapes chosen are common to the southeast USA. Extensive knowledge on the spatial and temporal availability and distribution of stink bugs and their parasitoids in non-crop and crop host plant habitats in farmscapes in southwest Georgia was utilized for strategic placement and timing of nectar provision for stink bug parasitoids. 

## 2. Field Studies on Nectar Provision for Stink Bug Parasitoids in Farmscapes

### 2.1. Milkweed Nectar for T. pennipes in Peanut-Cotton Farmscapes

For farmscapes in this region, adult food is lacking for *T. pennipes* either temporally or spatially. In the laboratory, feeding on raisins increased longevity and fecundity of *T. pennipes pilipes* F. by approximately 300% over water alone [62]. Coombs [63] studied the influence of adult food deprivation on longevity and fecundity of *T. giacomellii*. Females fed raisins had a mean longevity of 9.6 days, but survived only a mean of 3.2 days when provided with only water. Females given only water produced approximately 20% of the eggs of females with raisins as a food source. Clearly, availability of adult food can play an important role in the survival and reproduction of *Trichopoda* species. 

**Figure 1:**
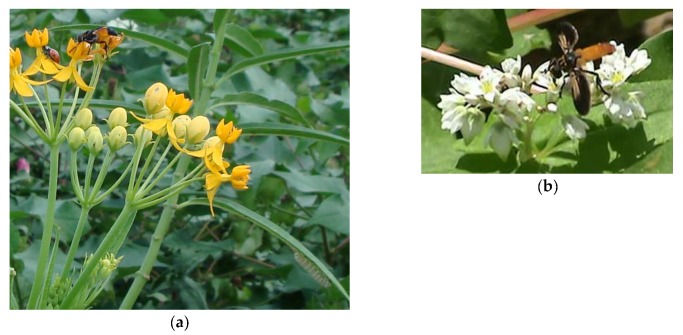
Nectar-provision: (**a**) *Trichopoda pennipes* and ladybeetle adult feeding on milkweed nectar with a monarch butterfly larva feeding on leaf; (**b**) *Trichopoda pennipes* feeding on buckwheat nectar.

Milkweed nectar is very rich in sugar, and the supply is renewed over the life of the individual flower [64,65]. The flowers of milkweed species are attractive to butterflies, bees, and other insect pollinators, as well as hummingbirds, and they provide a rich supply of nectar to these pollinator species [66,67]. Thus, tropical milkweed, *Asclepias curassavica* L., was placed in a corn-peanut-cotton farmscape to examine attractiveness and nectar-feeding of stink bug parasitoids and other insects in the field [68]. Each week throughout the growing season, every plant was observed for 2 min. on an hourly basis throughout the day. Although flowers are generally selected to benefit parasitoids, and not pests of the crop, flower morphology has been recognized as an important factor impacting accessibility of nectar to parasitoids, and thus should be taken into account [3]. Stink bug adult parasitoids, including *T. pennipes*, *C. binotata*, *E. tentatrix*; and egg parasitoids, *T. basalis* and *T. podisi*, though, regularly visited tropical milkweed and fed on its nectar (Figure 1a). Pollinators, including honey bees, *Apis mellifera* L., native insect pollinators, i.e., free-living flies and wasps and native bees, and parasitoids of lepidopteran pests, such as *Toxoneuron nigriceps* (Viereck) (Hymenoptera: Braconidae), also fed on tropical milkweed nectar. The monarch butterfly, *Danaus plexippus* (L.), of North America is renowned for its long-distance seasonal migration and its spectacular winter gatherings in Mexico and California. Monarch larvae feed exclusively on milkweeds in North America [69] (Figure 1a). In Georgia farmscapes, adult monarch butterflies fed on tropical milkweed nectar, and monarch larvae feeding on milkweed vegetation successfully developed into pupae [68].

Next, an experiment was conducted to determine if incorporation of tropical milkweed enhanced the biocontrol of stink bugs in plots in a peanut-cotton farmscape [70]. The two treatments included plots with potted milkweed plants placed between peanut and cotton along the interface of the two crops and plots without milkweed. Pesticides were not applied to the milkweed insectary. In the first year of the study, *N*. *viridula* was the primary host of *T. pennipes* in cotton, and parasitism of this pest by the parasitoid was significantly higher in milkweed cotton (61.6%) than in cotton alone (13.3%). In the second year of the study, parasitism of *N. viridula*, *C. hilaris*, and *Leptoglossus phyllopus* (L.) by *T. pennipes* was increased by approximately 25% when milkweed was present near cotton. For both years of the study, these treatment differences were not due to a response by the parasitoid to differences in host density, because density of hosts was similar for the two treatments. Milkweed was not a host plant for stink bugs, and adult lepidopteran pests were not observed feeding on plant nectar. In conclusion, incorporation of tropical milkweed at peanut-cotton interfaces increased stink bug parasitism in cotton and provided nectar to insect pollinators and monarch butterflies.

### 2.2. Buckwheat Nectar for T. pennipes in Peanut-Cotton Farmscapes

Flowers of buckwheat secrete nectar composed of sucrose, fructose, and glucose [71]. Nectar production attracts numerous parasitoid species, as well as insect pollinators [72], and the nectar is relatively accessible to parasitoids [73,74]. Another benefit of using buckwheat for nectar provision is that it is easy to establish [72]. Parasitoids exhibit enhanced performance when fed nectar of buckwheat. In the laboratory, buckwheat nectar increased the longevity of *Microplitis croceipes* (Cresson) at least 2-fold relative to wasps provided with only water [75]. Similarly, longevity of *D. insulare* feeding on buckwheat nectar was approximately two weeks longer than when feeding on water alone [74].

Because stink bugs exhibit edge-mediated dispersal at crop-to-crop interfaces as they colonize cotton, strategic placement of physical barriers, either synthetic or plant-based, at these interfaces can manage these pests. For both years of a field study, sorghum-sudangrass (*Sorghum bicolor* (L.) Moench × *S. bicolor* var. *sudanese*) and a 1.8-m-high polypropylene barrier wall effectively deterred dispersal of stink bugs into cotton [76]. Economic threshold was not reached in cotton for any of these treatments except for cotton with no barrier. In one year of the study, buckwheat was tested as a plant-based barrier. Pesticides were not applied to the buckwheat. Sorghum-sudangrass and buckwheat did not serve as host plants for stink bugs, and adult lepidopteran pests were not observed feeding on plant nectar. The stink bug adult parasitoid *T. pennipes* actively fed on buckwheat nectar (Figure 1b). Pollinators, including honey bees, *Apis mellifera* L., native insect pollinators, i.e., free-living flies and wasps and native bees, and *T. nigriceps* also fed on buckwheat nectar. Flowering buckwheat increased parasitism of *N. viridula* by *T. pennipes* by approximately 20% in nearby cotton even though it did not deter dispersal of stink bugs. Similarly, incorporating buckwheat in cabbage increased parasitism by *Voria ruralis* (Fallen) (Diptera: Tachinidae) on cabbage looper [*Trichoplusia ni* (Hübner)] (Lepidoptera: Noctuidae) larvae over a 4-yr study [15]. Further, the value of buckwheat flower strips has been demonstrated in apple orchards, where inter-sowing buckwheat increased parasitism by *Dolichogenidea tasmanica* Cameron (Hymenoptera: Braconidae) on the lightbrown apple moth, *Epiphyas postvittana* (Walker) [77]. Although not tested, the two management tactics, a plant-based physical barrier and incorporation of a nectar-producing plant, should be highly compatible with each other, and together could perhaps manage stink bugs and conserve insect pollinators by providing floral resources and eliminating or reducing insecticide applications.

### 2.3. Nectar Provision for T. podisi in a Soybean Trap Cropping System 

One strategy for managing dispersing insect pests is trap cropping where a preferred plant species is used to arrest pests and reduce their likelihood of entering a crop [78]. In cotton-soybean farmscapes, stink bugs are known to prefer fruiting soybean to fruiting cotton [23]. Thus, a study was conducted to examine the ability of a strip of soybean placed between peanut and cotton field plots to deter stink bugs from colonizing cotton [79]. An earlier study revealed that the stink bug egg parasitoid *T. basalis* lived longer when females had access to floral nectaries of nine plant species, including *Tagetes patula* L. and buckwheat [80]. Therefore, a strip of flowering buckwheat was planted alongside soybean in some plots to examine the influence of nectar-provision on parasitism of sentinel *E. servus* egg masses in the cotton row closest to soybean [79]. Multiple planting dates for buckwheat ensured continuous flowering while soybean and cotton were fruiting. Pesticides were not applied to the trap cropping system.

Soybean was an effective trap crop for *C. hilaris*, *E. servus*, and *N. viridula*, reducing both stink bug density in cotton and boll injury regardless of whether it was used alone or in combination with buckwheat [79]. Incorporation of buckwheat into the trap cropping system, though, provided an additional ecosystem benefit by enhancing parasitism of *E. servus* egg masses by *Telenomus podisi* Ashmead. Recently, Lahiri et al. [81] reported enhanced longevity and fecundity of *T. podisi* when fed with buckwheat nectar under laboratory conditions. The study showed that buckwheat nectar is as effective at benefitting *T. podisi* as pure honey, providing evidence that buckwheat is a nutritionally suitable food source for this parasitoid. Parasitism of *E. servus* eggs in cotton near buckwheat-soybean plots was moderate, 40%. These results are in agreement with other studies using buckwheat for nectar provision in the field [15,16,77]. Leafroller parasitoids caught in yellow sticky traps increased in number when buckwheat flowers were present in a vineyard [82]. Thus, parasitism of *E. servus* eggs by *T. podisi* might be further enhanced by providing flowering buckwheat earlier in the season to increase abundance of well-fed parasitoids as stink bugs disperse and aggregate in soybean. Pease and Zalom [39] evaluated stink bug egg parasitism of *E. conspersus* sentinel egg masses in fresh market tomatoes adjacent to a sweet alyssum (*Lobularia maritima* L.) border with an unplanted control border at three sites. Egg parasitism by scelionid species was significantly greater in tomatoes with an alyssum border, but only late in the season. The authors suggested that an earlier planting of alyssum might enhance parasitism earlier in the season.

Molecular gut-content analysis based on polymerase chain reaction (PCR) was used to detect a complex of stink bug DNA in a complex of predators in soybean and cotton in the above study [83]. Detection of the remains of crop-specific prey in predators’ guts demonstrated predator dispersal between soybean and cotton. Combined density of the predators *Geocoris punctipes* (Say) and *G. uliginosus* (Say) was higher in soybean with buckwheat than in soybean alone, indicating that they were attracted to, and maybe feeding on, buckwheat nectar. DeLima and Leigh [84] reported that nectar is essential for development of *Geocoris pallens* Stål on cotton in the absence of prey. Similarly, density of the predator *Jalysus wickhami* VanDuzee (Hemiptera: Berytidae) was significantly greater in tomatoes with alyssum than tomatoes without this nectar-producer [39]. The percentage of *Geocoris* spp. screening positive for stink bug DNA was high, 87.3%, for *N. viridula*, moderately high, 60.3%, for *E. servus*, and relatively low, 29.6%, for *C. hilaris* in soybean [83]. A combination of a nectar-producing plant with a trap crop or plant-based physical barrier can also serve as a refuge for predators of stink bugs which can forage between the refuge and the cash crop, likely enhancing predation, as well as parasitism of stink bugs.

## 3. Discussion

Providing nectar-producing plants in peanut-cotton farmscapes enhanced parasitism of multiple stink bug species by an adult and egg parasitoid in southwest Georgia. The successful increase in biocontrol of stink bug hosts in cotton was due to a combination of factors. The nectar-producing plants utilized were optimal as parasitoid food sources, for they combined attractiveness with accessible nectar. Additionally, the approach was designed to place nectar-producing plants along the crop-to-crop interface at a time when stink bugs were colonizing cotton at this interface. Strategic spatial and temporal arrangement of nectar-producing plants is applicable to other insect pests in their particular agroecosystems [85]. Parasitism of the bagworm, *Thyridopteryx ephemeraeformis* (Haworth) by a guild of parasitoids exceeded 70% in shrubs that were adjacent to a central bed of flowering forbs, but less than 40% in shrubs that were farther away [86]. Similarly, *D. semiclausum* adults were capable of moving over distances of 80 m, but they were more effective as biological control agents at 60 m; the spatial scale at which floral resources were available [87]. Parasitism was greater in *P. operculella* larvae recovered from potato plants growing close to a strip of flowers than in larvae 20 m distant, and parasitism rates declined as the distance from floral resources increased [14]. Each of these studies attests to the importance of considering the spatial influence of floral resources on natural enemies. Wäckers [88] and Takasu and Lewis [89] demonstrated that sugar deprivation reduces host searching efficiency due in part to spending more time searching for hosts than searching for food. It is imperative that floral resources are provided in close proximity to hosts to enhance searching efficiency of parasitoids. An extension of flowering buckwheat from a patch of a non-crop host and then along a crop-to-crop interface in farmscapes with closely associated crops could possibly serve as temporal and spatial bridge for natural enemy entrance into the cotton field. Nicholls et al. [90] monitored distribution and abundance patterns of pests and natural enemies in two monoculture vineyard blocks. One block was cut across by a corridor composed of 65 flowering species that was connected to a riparian forest. The presence of riparian habitats enhanced predator colonization and abundance of adjacent vineyards. The corridor amplified this influence by allowing enhanced and timely circulation and dispersal movement of predators into the center of the field. 

Incorporation of nectar producing plants could perhaps enhance parasitism of *H. halys*, as well as native species, by native parasitoid species. Future research will be conducted in locations in central Georgia where *H. halys* has become established to test this hypothesis. Recently, the Asian egg parasitoid *Trissolcus japonicus* (Ashmead) was discovered parasitizing *H. halys* eggs in a woodland habitat [91]. Pickett et al. [92] used buckwheat as an insectary to introduce *T. pennipes* for control of the squash bug. Perhaps incorporation of buckwheat, or other nectar-producing plants, could enhance establishment of *T. japonicus* in a classical biological control program targeting *H. halys*.

Several studies have demonstrated increased parasitism or predation by provision of nectar-producing flowers in an agroecosystem, but only a few studies have shown nectar provision to reduce pest damage. In one such study, local nectar-producing plants were grown around rice fields in multiple sites in 3 countries [93]. This inexpensive tactic reduced the abundance of two key pests, reduced insecticide applications by 70%, increased rice yields by 5%, and resulted in an economic advantage of 7.6%. Unfortunately, in many cases, parasitism of the insect pest may not lead to significant or consistent reduction in crop damage, because the parasitized pest continues to feed on the crop. Addition of cornflowers (*Centaurea cyanus* L.) into cabbage fields increased larval parasitism of *Mamestra brassicae* (L.) by *Microplitis mediator* (Haliday) and egg parasitism and predation of the herbivore, reduced crop damage, and increased crop yield for one or two years [94]. The authors suggested that egg parasitoids or predators may be the best target for nectar provision, for a parasitized larva continues feeding on the crop as the parasitoid develops in its host. Nectar-provision, though, can be compatible with other management tactics, i.e., trap cropping and physical barriers, to deter dispersal and oviposition in a cash crop to manage insect pests below economic thresholds. Mizell et al. [95] developed a stink bug trap cropping system composed of sorghum and pearl millet, *Pennisetum glaucum* (L.) R.Br., and two nectar-producing plants, buckwheat and sunflower, *Helianthus annuus* L. This multifunctional habitat effectively managed *E. servus*, *C. hilaris*, and the leaffooted bug, *Leptoglossus phyllopus* (L.) in organically-grown soybean. In addition, both buckwheat and sunflower provided nectar to natural enemies and insect pollinators.

The value of nectar provision in agroecosystems might be magnified and more readily accepted by producers and the general public if additional ecosystem services such as conservation of insect pollinators (i.e., honey bees and native pollinators), natural enemies of pest insects, and iconic flora or fauna (e.g., the monarch butterfly) could be included. Partnerships are being encouraged to link conservation biological control with other activities that would strengthen other ecosystem services. For example, The Food, Conservation, and Energy Act of 2008 authorizes the United States Department of Agriculture (USDA) to promote the development of habitats to conserve native and managed pollinators on agricultural lands [96]. Honey bees play a critical role in the pollination of many agricultural crops. Colony collapse disorder (CCD), in which honey bee colonies inexplicably lose their workers, has resulted in a loss of 50 to 90% of colonies in beekeeping operations across the USA [97]. Bee declines are driven by combined stress from parasites, pesticides, and lack of flowers in intensively farmed areas and urban areas [98]. The monarch butterfly faces many threats, including reduction of native milkweed populations [69]. Because milkweed flowers provide a rich supply of nectar and bloom from spring through the fall in temperate zones, native milkweed species may be excellent choices for nectar provision for these honey bees and native insect pollinators in agricultural farmscapes, as well as conserving the monarch butterfly. Buckwheat is another excellent choice for conserving natural enemies of pest insects and insect pollinators by providing floral resources and eliminating or reducing insecticide applications.

## 4. Conclusions

Adult parasitoids require nectar for survival and oviposition. Parasitoids can be severely food-deprived in modern, intensively farmed agroecosystems lacking in floral resources. Provisioning nectar-producing plants in these agroecosystems has the potential to enhance biocontrol of insect pests. An understanding of the biology and ecology of insect pests and their parasitoids is necessary for designing an effective spatial and temporal arrangement of a nectar-provision habitat to improve biological control. The goal of this review was to present examples of on-farm application of nectar-provision for stink bug parasitoids. Stink bugs move between closely associated host plants throughout the growing season in response to the deteriorating suitability of their current host plants, and exhibit an edge effect in dispersal from one host plant to another. Strategic positioning and timing of nectar-producing plants, either milkweed or buckwheat, at these field edges in agroecosystems enhanced parasitism of stink bug adult and egg parasitoids. Moreover, the presence of flowering plants attracted insect pollinators such as honey bees, thus enhancing other ecosystems services (e.g., pollination), as well as conserving the endangered Monarch butterfly.
